# An Overview of Systematic Reviews of Randomized Controlled Trials on Acupuncture Treating Migraine

**DOI:** 10.1155/2019/5930627

**Published:** 2019-10-29

**Authors:** Xia-tian Zhang, Xin-yi Li, Chen Zhao, Ye-yin Hu, Yi-yi Lin, He-qing Chen, Zhao-feng Shi, Xiao-yu Zhang, Hong-cai Shang, Gui-hua Tian

**Affiliations:** ^1^Key Laboratory of Chinese Internal Medicine of MOE and Beijing, Dongzhimen Hospital, Beijing University of Chinese Medicine, Beijing 100700, China; ^2^Institute of Basic Research in Clinical Medicine, China Academy of Chinese Medical Sciences, Beijing 100700, China

## Abstract

**Objectives:**

To review the evidence of acupuncture for acute and preventive treatment of migraine for further awareness of the effect of acupuncture for migraine.

**Design:**

An overview of systematic reviews and meta-analyses (SR/MAs) for randomized controlled trials.

**Material and Methods:**

We searched PubMed, Embase, the Cochrane Library, China Knowledge Resource Integrated Database, VIP Chinese Journal Full Text Database, WANFANG Data, and China Biology Medicine disc from their establishment to May 27, 2018. SR/MAs of randomized controlled trials comparing the effect of the acupuncture intervention with another treatment control in migraine patients were included.

**Results:**

428 SRs were identified, and 15 of them were included. Only 4 SR/MAs were assessed by GRADE, which showed certainty of most evidence being low or very low. Assessed by AMSTAR-2, fourteen was critically low rating overall confidence in the results, and 1 was low rating overall confidence in the results. Evidence suggested that acupuncture has a significant advantage of pain improvement, efficacy, and safety relative to blank control, sham acupuncture, or drug treatment, but some of these results are contradictory.

**Conclusions:**

We found that acupuncture on treating migraine has the advantage for pain improvement and safety, but the quality of SR/MAs of acupuncture for migraine remains to be improved.

## 1. Introduction

Migraine is a common disabling primary headache disorder [1]. Epidemiological studies revealed that the global age-standardized prevalence of migraine was 14.4% (13.8–15.0%) in 2016, and this figure was 18.9% (18.1–19.7) for women and 9.8% (9.4–10.2) for men; in addition, the prevalence by ages increased significantly until reaching a peak between 35 and 39 years and decreased smoothly after 40 years, and for China, the prevalence of migraine was 9.3% (8.5–10.1%) in 2009 [2, 3]. Migraine has two major subtypes, “migraine without aura” and “migraine with aura,” and the mechanism of migraine is related to vascular, pain pathway, central system [1]. Acute treatments for migraine usually include using aspirin or triptans (a highly selective serotonin 5-HT_1B_ and 5-HT_1D_ receptor agonists), and other drugs, whereas preventive treatment adopts drugs, cognitive behavioral therapy, and other therapeutic methods [4, 5].

Acupuncture is a kind of physical therapy, which is an essential component of “Traditional Chinese Medicine” (TCM) [6]. TCM theory believes that a kind of critical energy called Qi maintains the regular operation of the human body and this energy flows in a network of channels called meridians. The theory of TCM also believes the human body will suffer from illness or disease once this flow is abnormal, which can be recovered by inserting the needle in some specified points on the channels of meridians of the human body like acupuncture. Acupuncture is often used to treat headache including migraine [7], and its therapeutic mechanism may be related to its regulation of nitric oxide synthase and 5-hydroxytryptamine (5-HT) gene_1F_ expression to improve cerebral vasodilation and contraction [8].

Systematic reviews can provide a robust review of the effectiveness of clinical interventions [9]; according to the literature search, clinical researchers have completed a number of systematic reviews of acupuncture treating migraine. To summarize the evidence of acupuncture for migraine [10] and show the effect of acupuncture for acute and preventive treatment of migraine, the researchers carried out an overview of systematic reviews/meta-analyses (SR/MA) of randomized controlled trials (RCTs) on acupuncture for migraine.

## 2. Material and Methods

### 2.1. Studies Searches

We searched PubMed, Excerpta Medica database (Embase), the Cochrane Library, China Knowledge Resource Integrated Database, VIP Chinese Journal Full Text Database, WANFANG Data, and China Biology Medicine disc from their establishment to May 27, 2018, with the following search terms: “migraine^*∗*^,” “status migrainosus,” “sick headache,” “acupuncture,” “needle,” “needling,” “thorns,” “dry-needling,” “body-acupuncture,” “stitch,” “tapping,” “electroacupuncture,” “electro-acupuncture,” “prick,” “pricking,” “bloodletting,” “puncturing collateral,” “bleeding therapy,” “Acusector,” “quick puncture,” “blood-letting,” “systematic review,” “SR,” “systematic evaluation,” “systematic assessment,” “meta-analysis^*∗*^,” and “Cochrane review.”

### 2.2. Inclusion and Exclusion Criteria

The inclusion criteria were as follows: ① Type of studies: SR/MAs of randomized controlled trials, ② Type of participants: migraine diagnosed by any internationally recognized or accepted clinical guideline or consensus like *The International Classification of Headache Disorders*, *3rd edition (beta version)* [1], ③ Type of interventions: the experimental groups were treated with acupuncture, which contained acupuncture with electrical stimulation and other acupuncture techniques with needles inserted into the skin, ④ Type of control: the control groups were treated with other blank controls, placebo, drug treatments, or other TCM treatments ⑤ Type of Outcomes: the primary outcomes were as follows: (a) pain intensity rated by measure tools: visual analogue scales (VAS), numerical rating scales (NRS) score, and verbal rating scale (VRS) score; (b) usage of (rescue) analgesics (any continuous or rank measures available), which was recommended by *Initiative on Methods*, *Measurement*, *and Pain Assessment in Clinical Trials* [11]; and (c) severe events; secondary outcome was frequency of migraine attacks (per 4 weeks or per month), headache frequency or times (per 4 weeks or per month), the number of migraine days (per 4 weeks or per month), the number of headache days (per 4 weeks or per month), and effective rate (curing rate or improving rate evaluated by researchers or subjects of studies included), which is lack of international recognition but commonly used in some Chinese literature. With the following situations, the studies were excluded: ① non-SR/MA, ② non-Chinese and English studies, and ③ no full-text studies were available.

### 2.3. Data Extraction

All searched studies were imported into Endnote X8.0 for document management. Two coauthors (YH, HC) screened the titles and abstracts of all studies according to the inclusion and exclusion criteria, and then full texts of possible relevant studies were screened. Any disagreement was resolved by consulting another coauthor (XL).

EpiData 3.1 was used to extract the following items data by three coauthors (YL, HC, and XL): ① Basic information of included SR/MAs: the number of studies, interventions, and main conclusions; ② Methodology and evidence certainty of included SR/MAs: methodological information of SR/MA that recorded in *AMSTAR-*2 (a measurement tool to assess systematic reviews) [12], certainty of evidences reviewed by *GRADE* (Grading of Recommendations, Assessment, Development, and Evaluations) [13]; ③ Effect of intervention: outcomes of SR/MAs.

### 2.4. Assessment of Methodology of Included Reviews

AMSTART-2 was used to assess the methodological quality of included SR/MAs: When no or only 1 nonkey items did not conform, inferring rating overall confidence in the results of the SR/MA as *high*; when more than 1 nonkey item did not conform, inferring rating overall confidence in the results of the SR/MA as *moderate*; when 1 key item did not conform with nonkey items conforming or not conforming, inferring rating overall confidence in the results of the SR/MA as *low*; and when more than 1 key items did not conform with nonkey items conforming or not conforming, inferring rating overall confidence in the results of the SR/MA as *critically low*.

### 2.5. Statistical Analysis

We use frequency to show data (types of included studies' therapies, types of outcome indexes, AMSTAR-2 compliance items, outcome indexes of meta-analysis, meta-analysis results, and so on). Microsoft Excel 365 was used for data visualization: Reformat data extracted from the underlying systematic reviews in tables.

According to SR/MAs, the results of quantitative synthesis were reported in the form of standard mean deviation (SMD), weighted mean deviation (WMD), odds ratio (OR), or relative risk (RR). And the results were also reported with 95% confidence intervals (CI).

## 3. Results

### 3.1. Study Selection Process

**Figure 1:**
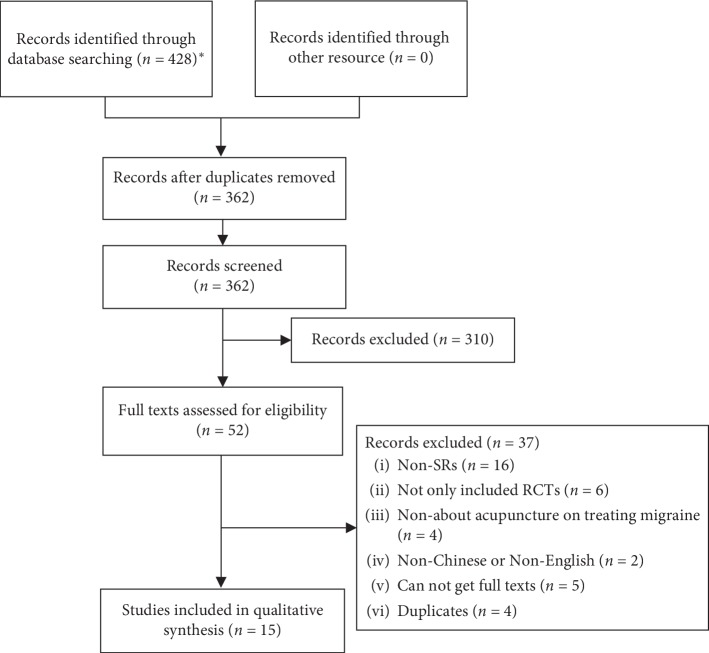
Studies selection for acupuncture treating migraine SR/MAs flow diagram.

A total of 428 studies were initially searched. 52 studies were selected after reading the title and abstract, and 15 SR/MAs were included in the overview after viewing full texts [7, 14–27], which included 2 Cochrane systematic reviews (9.5%) [7, 14] and 19 non-Cochrane system reviews (90.4%). The study selection process is shown in Figure 1.

### 3.2. Study Characteristics

The basic information of the included studies is presented in Table 1. For the 15 SR/MAs, 13 studies included migraine patients [14–22, 24–27], 1 included episodic migraine patients (participants had been diagnosed with episodic migraine) [7] and one study included menstrual migraine patients (participants had been diagnosed by *the International Classification of Headache Disorders s-1/2/3* or criteria of menstrual migraine in *Criteria for the Diagnosis and Therapeutic Effect of Diseases and Symptoms in Traditional Chinese Medicine* [30] published by the State Administration of Traditional Medicine of China) [23]. For the timing of intervention, 11 studies did not define acute or preventive treatment, whereas 1 study was for acute treatment [25] and 3 studies were for preventive treatment [7, 24, 26].

### 3.3. Certainty of Evidences of Systematic Reviews Included

In the certainty of the evidence, only 4 studies were reviewed by *GRADE* [7, 16, 23, 24], the certainty of the evidence of these studies was like Table 2.

### 3.4. Methodological Quality

In the aspect of methodological quality, no studies were all conformed, and one was *low* rating overall confidence in the results [7], and fourteen were *critically low* rating overall confidence in the results [14–27], like Table 3.

### 3.5. Meta-Analyses Outcomes of Intervention

#### 3.5.1. Outcomes of Pain Improvement

For the *VAS score*, 2 SRs reported it as meta-analyses (MAs) outcomes like Table 4 [18, 25]. For the headache situation, 5 SRs reported it as MAs outcomes like Table 5 [7, 14, 20, 24, 26].

#### 3.5.2. Outcomes of Efficacy

For efficacy, 12 SRs reported it as MAs outcome indexes like Table 6 [7, 14–17, 19, 20, 22–24, 26, 27].

#### 3.5.3. Outcomes of Safety

For adverse events, 9 SR/MAs reported adverse events [7, 14–16, 21, 23, 24, 26, 27], but only 4 reported it as MAs outcome indexes like Table 7 [7, 14, 16, 24].

## 4. Conclusions of SR/MAs Included

In terms of conclusion, the results of all 15 (100%) SR/MAs were positive. For treatment, 6 SR/MAs reported acupuncture had superiority relative to drugs [7, 16, 17, 19, 26, 27]; 4 SR/MAs reported acupuncture had superiority relative to sham acupuncture, drugs [14, 18, 22, 24]; 3 SR/MAs reported acupuncture had superiority relative to sham acupuncture [20, 21, 25]; 1 SR/MA reported acupuncture had superiority relative to drugs, other TCM treatments [16]. 1 SR/MA reported that acupuncture had superiority in treating migraine, but did not mention the control group in the conclusions [23].

## 5. Discussions

### 5.1. Summary of Main Findings

This overview included a considerable number of SR/MAs, illustrating that acupuncture has the advantage in pain improvement of VAS score, headache days/frequency, analgesic use and efficacy of response rate, and effective rate according to the present evidence. However, most SR/MAs included did not conclude firmly because of the small size or low methodological quality of the included trials and subjectively evaluated outcome of included trials, and some SR/MAs also stated that high-quality acupuncture for migraine RCT was still needed to confirm the main findings further. Based on previous evidence we reviewed, we supposed acupuncture might be a kind of available treatment for migraine in preventive or acute treatment, but in consideration of low methodological quality of present SR/MAs or RCTs of acupuncture for migraine, we need more high-quality evidence to demonstrate the effect of acupuncture for migraine.

### 5.2. Designs of Present SR/MAs

There was some advice for later researchers to carry out high-quality SR/MAs or RCTs of acupuncture for migraine, which was summarized according to designs of present SR/MAs.

### 5.3. Participants

Most studies included did not mention whether the patients they included were with acute attacks of migraines or not, which reduce the significance of most included SR/MAs in revealing the suitable timings of applying acupuncture for migraines. Therefore, later researchers can get more meaningful results with a more clear statement of patients' conditions like whether they are with aura or attacks.

### 5.4. Control Group

The control interventions included in SR/MAs were blank control, sham acupuncture, and drug treatment. In addition, one of the heterogeneity sources may be regarding different types of drug treatment as 1 kind of subgroup. As a result, later researchers should provide a clear comparison of intervention vs. control in their studies, which can not only improve the robustness of results but also offer suggestions of combined therapy including acupuncture in clinical practice.

### 5.5. Outcome

The most common outcomes were about an effective rate. And results of SR/MAs in most outcomes were contradictory, which means we inferred that the efficacy is evaluated by both patient's own pain feeling and the estimate of different doctors; therefore, a certain degree of subjectivity in evaluation might appear, hence the difference in medical sets, communicating skills of doctors or other factors in each SR/MA, which can lead to bias in the conclusions of SR/MAs. We consider that SR/MAs and RCTs of acupuncture for migraine have problems with outcome indexes, for instance, outcomes were difficult to be evaluated stably and were also not international recognized like other clinical studies of other TCM treatments [31]. So, later researchers can also increase the reliability of their studies by regarding international recognized outcomes as outcomes in their studies.

### 5.6. Methodological Quality and Evidence Certainty of Systematic Reviews Included

Researchers of included SR/MAs generally concluded that the reliability of their own SR/MAs evidence was low, more high-quality, large sample of acupuncture treating migraine RCT trials were needed, and the low quality of acupuncture treating migraine may affect the reliability of the evidence of SR/MAs in acupuncture for migraine. In addition, this study suggests that some researchers of SR/MAs did not synthesize data of different follow-up time and whether were grouped with acute attacks or not, respectively, which may also affect the heterogeneity of results provided by SR/MAs and reduce the certainty of their studies. And there were only 4 studies using *GRADE* to review the certainty of the evidence, whereas most meta-analysis outcomes were low or very low certainty. As assessment of *AMSTAR-2*, SR/MAs in acupuncture treating migraine need to be improved in methodology and reporting quality as these points below: lack of research protocol statement and registration may affect the transparency of the research results, lack of showing and explaining the reason of excluding studies may leave some information missing, lack of revealing funding sources of included studies may make researchers or readers ignore potential benefits and conflicts, and lack of statement of self-interest relationship may make readers ignore factors affecting objectivity and reliability of SR/MAs.

The core reason of low methodological quality of SR/MAs may be that a large portion of researchers of SR/MAs in acupuncture for migraine may not receive standard evidence-based medical education; therefore, we should strengthen and improve the work of evidence-based medicine education in higher education and continuing education of TCM, especially with the selection of suitable outcomes.

### 5.7. Strength and Limitations

The strengths of this study were as follows: ① both English and Chinese studies were included to promise included studies widely and ② each MAs outcome was showed in structured tables which can help readers realize or review interesting outcomes easily. The limitations of this study were as follows: ① SR/MAs included were all low quality, which may reduce the confidence of the results, and ② the conflicts of interest of SR/MAs included were not analyzed, which may induce to miss some information.

## 6. Conclusions

From this study, we found that acupuncture has the advantage for acute and preventive treatment of migraine in pain improvement and safety, but the quality of SR/MAs of acupuncture for migraine still needs to be improved.

## Figures and Tables

**Table 1:** Basic information of included studies.

Study ID	Participant	Acute or preventive treatment	Intervention	Control	Outcome measures	Design	Trials (participants)	Risk of bias assessment tool
Linde et al. [14]	Migraine patients	NR	Acupuncture	①②③	At least one clinical outcome related to headache	RCT	26 (1151)	Risk of bias approach for Cochrane reviews
Linde et al. [7]	Episodic migraine patients	Preventive treatment	Acupuncture with or without manual or electrical stimulation	①②③	Headache frequency, response, and quality of life	RCT	22 (4985)	*α*
Chen et al. [15]	Migraine patients	NR	Acupuncture	③	Ⓑ	RCT	18 (1730)	*β*
Chen [16]	Migraine patients	NR	Acupuncture	Acupuncture not according to *meridians*;①②③ and other treatments	Ⓑ	RCT	18 (1672)	*α*
Chu et al. [17]	Migraine patients	NR	Acupuncture	②③	NR	RCT	24 (2397)	*α*
Cui et al. [18]	Migraine patients	NR	Acupuncture with or without electrical stimulation	②③ and other treatments.	Ⓐ	RCT	15 (1288)	*α*
Dai and Lin [19]	Migraine patients	NR	Acupuncture	NR	NR	RCT	2 (140)	*β*
Gao et al. [20]	NR	NR	Acupuncture	②	Ⓑ	RCT	12 (1744)	*β*
Yang et al. [21]	Migraine patients	NR	Acupuncture	②	At least one clinical outcome related to headache	RCT	10 (997)	*α*
Zheng and Hai [22]	Migraine patients	NR	Acupuncture	NR	NR	RCT	33 (3593)	*α*
Zhao [23]	Menstrual migraine patients	NR	Acupuncture with or without other treatments	②③ and other treatments.	ⒶⒷⒸ, pain index, pain time, symptom score, and biochemical indexes.	RCT	18 (1268)	*α*
Pu [24]	Migraine patients	Preventive treatment	Acupuncture	③	ⒶⒷⒸ, migraine days, migraine frequency, times of analgesics used, and follow-up rate.	RCT	7 (1285)	*α*
Pu et al. [25]	Migraine patients	Acute treatment	Acupuncture	②	Ⓐ	RCT	5 (618)	*α*
Song et al. [26]	Migraine patients	Preventive treatment	Acupuncture with or without electrical stimulation or placebo	③ with or without placebo	Ⓑ, pain days, and other outcome.	RCT	18 (1470)	*α*
Yang et al. [27]	Migraine patients	NR	Acupuncture	③	ⒷⒸ; pain index	RCT	10 (893)	*α*

^*∗*^NR: not reported in inclusion criteria; ①: no acupuncture; ②: sham acupuncture; ③: drug treatment; Ⓐ: visual analogue scale (VAS) pain score; Ⓑ: efficacy: Ⓒ: adverse events; *α*: Cochrane risk of bias assessment tool [28]; *β*: Jadad scale [29].

**Table 2:** Certainty of evidences reviewed by *GRADE*.

Study ID	Intervention	Control	Outcomes	Certainty of evidences
Linde et al. [7]	Acupuncture	No treatment/usual care	Headache frequency (after treatment) assessed with days per month; response (after treatment and follow-up) assessed with proportion of participants with at least 50% headache frequency reduction	Moderate
			Headache frequency (follow-up) assessed with days per month	Low
	Acupuncture	Sham acupuncture	Headache frequency (after treatment and follow-up) assessed with days per month; response (after treatment and follow-up) assessed with proportion of participants with at least 50% headache frequency reduction	Moderate
			Number of participants dropping out because of adverse effects	Low
			Number of participants reporting adverse effects	High
	Acupuncture	Prophylactic drug treatment	Headache frequency (after treatment and follow-up) assessed with days per month; response (after treatment and follow-up) assessed with proportion of participants with at least 50% headache frequency reduction; number of participants dropping out because of adverse effects; number of participants reporting adverse effects	Moderate

Chen [16]	Acupuncture	Drug treatment	Effective rate	Low∼moderate
			Headache frequency, headache intensity, length of headache, combined symptoms, and symptom score	Moderate
	Acupuncture	Other TCM treatment	Effective rate, headache frequency, and combined symptoms	Low
			Headache intensity and length of headache	Very low
	Acupuncture	NR	VAS score and adverse events	Moderate
			TCD-MCA;TCD-ACA; TCD-PCA;TCD-VA	Low
			TCD-BA	Very low

Zhao [23]	Acupuncture	Drug treatment	Effective rate, pain index, headache intensity, length of headache, combined symptoms score, and symptom score	Very low
	Acupuncture with acupuncture on ear	Drug treatment	Effective rate of mild patients	Low
			Effective rate, effective rate of moderate patients, effective rate of severe patients, headache score, *β*-EP, Vasopressin, NO, PGF, ET-1, and adverse effects.	Very low
	Acupuncture with TCM drug treatment	Drug treatment	Effective rate	Very low
	Acupuncture with electrical stimulation	Drug treatment	Effective rate	Very low
	Acupuncture for bloodletting	TCM drug treatment	Effective rate, headache intensity, and length of headache	Very low

Pu [24]	Acupuncture	Drug treatment	Migraine days (5-6 months); migraine times (3-4 months); effective rate (5-6 months)	Moderate
			Migraine days (3-4 months), migraine times (5-6 months), headache intensity (5-6 months), and number of use of analgesics (5-6 months)	Low
			Headache intensity (3-4 months), number of use of analgesics (3-4 months), effective rate (3-4 months), adverse effects, and follow-up rate	Very low

^*∗*^TCD: transcranial doppler; MCA: middle cerebral artery; ACA: anterior cerebral artery; posterior cerebral artery: VA: vertebral artery; BA: basilar artery; *β*-EP: beta-endorphin; NO: nitric oxide; PG: prostaglandin; ET: endothelin.

**Table 3:** AMSTAR-2 assessment table.

AMSTAR-2 items	Yes		Partially yes		No	
	*n*	%	*N*	%	*n*	%
A01	12	80.0	0	0.0	3	20.0
A02	0	0.0	0	0.0	15	100.0
A03	15	100.0	0	0.0	0	0.0
A04	0	0.0	14	93.3	1	6.7
A05	12	80.0	0	0.0	3	20.0
A06	12	80.0	0	0.0	3	20.0
A07	2	13.3	1	6.7	12	80.0
A08	4	26.7	8	53.3	3	20.0
A09	12	80.0	3	20.0	0	0.0
A10	0	0.0	0	0.0	15	100.0
A11	15	100.0	0	0.0	0	0.0
A12	9	60.0	0	0.0	6	40.0
A13	10	66.7	0	0.0	5	33.3
A14	9	60.0	0	0.0	6	40.0
A15	9	60.0	0	0.0	6	40.0
A16	1	6.7	0	0.0	14	93.3

^*∗*^A01: did the research questions and inclusion criteria for the review include the components of PICO? A02: did the report of the review contain an explicit statement that the review methods were established prior to the conduct of the review and did the report justify any significant deviations from the protocol? A03: did the review authors explain their selection of the study designs for inclusion in the review? A04: did the review authors use a comprehensive literature search strategy? A05: did the review authors perform study selection in duplicate? A06: did the review authors perform data extraction in duplicate? A07: did the review authors provide a list of excluded studies and justify the exclusions? A08: did the review authors describe the included studies in adequate detail? A09: did the review authors use a satisfactory technique for assessing the risk of bias (RoB) in individual studies that were included in the review? A10: did the review authors report on the sources of funding for the studies included in the review? A11: if meta-analysis was performed, did the review authors use appropriate methods for statistical combination of results? A12: if meta-analysis was performed, did the review authors assess the potential impact of RoB in individual studies on the results of the meta-analysis or other evidence synthesis? A13: did the review authors account for RoB in individual studies when interpreting/ discussing the results of the review? A14: did the review authors provide a satisfactory explanation for, and discussion of, any heterogeneity observed in the results of the review? A15: if they performed quantitative synthesis, did the review authors carry out an adequate investigation of publication bias (small study bias) and discuss its likely impact on the results of the review? A16: did the review authors report any potential sources of conflict of interest, including any funding they received for conducting the review?

**Table 4:** MAs outcomes about VAS score.

Study ID	Outcome	Acute or preventive treatment	Intervention	Control	Follow-up time	RCTs (*n*)	I2	Model	MD	95% CI	*P* value
Cui et al. [18]	VAS score	ND	Acupuncture with or without electrical stimulation	① or ②	Immediate-term (ND)	5 (667)	87%	REM	−0.32	[−1.04, 0.41]	<0.0001
			Acupuncture		Short-term (ND)	7 (447)	66%	REM	−1.24	[−1.84, −0.64]	<0.0001
					Long-term (ND)	5 (318)	95%	REM	−1.77	[−2.76, −0.78]	0.0005

Pu et al. [25]	VAS score	Acute treatment	Acupuncture	①	2 h	4 (699)	67%	REM	−0.38	[−0.83, 0.07]	0.10
					4 h	4 (699)	70%	REM	−0.42	[−0.96, 0.12]	0.12
	VAS score reduction				2 h	3 (579)	0%	FEM	0.36	[0.08, 0.65]	0.01
					4 h	3 (579)	0%	FEM	0.49	[0.14, 0.84]	0.01

^*∗*^ND: not defined; ①: sham acupuncture; ②: drug treatment.

**Table 5:** MAs outcomes about headache situation.

Study ID	Outcome	Acute or preventive treatment	Intervention	Control	Follow-up time	RCTs (*n*)	I2	Model	Scale	Effect	95% CI	*P* value
Linde et al. [14]	Headache frequency	NR	Acupuncture	①	3-4 months after randomization	4 (2087)	44%	REM	SMD	−0.43	[−0.60, −0.27]	<0.00001
	Headache days				3-months after randomization	3 (2064)	12%	REM	MD	−2.09	[−2.60, −1.58]	<0.00001
	Analgesic use				2 months after randomization	2 (218)	0%	REM	SMD	−0.29	[−0.59, 0.01]	0.057
					3-4months after randomization	4 (581)	92%	REM	SMD	−0.52	[−1.22, 0.18]	0.15
	Headache frequency			②	2 months after randomization	6 (895)	57%	REM	SMD	−0.23	[−0.49, 0.04]	0.09
					3-4months after randomization	8 (1012)	57%	REM	SMD	−0.18	[−0.44, 0.07]	0.16
					5-6 months after randomization	5 (932)	55%	REM	SMD	0.01	[−0.25, 0.28]	0.92
					>6 months after randomization	4 (150)	0%	REM	SMD	0.11	[−0.22, 0.43]	0.52
	Migraine attacks				2 months after randomization	4 (327)	21%	REM	MD	−0.35	[−0.74, 0.04]	0.079
					3-4 months after randomization	5 (360)	40%	REM	MD	−0.11	[−0.51, 0.30]	0.61
					5-6 months after randomization	4 (321)	51%	REM	MD	0.15	[−0.36, 0.65]	0.57
					>6 months after randomization	4 (150)	0%	REM	MD	0.30	[−0.10, 0.71]	0.14
	Migraine days				2 months after randomization	5 (864)	65%	REM	MD	−0.80	[−1.70, 0.11]	0.084
					3-4 months after randomization	6 (951)	52%	REM	MD	−0.27	[−0.98, 0.45]	0.46
					5-6 months after randomization	5 (928)	37%	REM	MD	−0.17	[−0.82, 0.47]	0.60
					>6 months after randomization	4 (140)	0%	REM	MD	0.35	[−0.90, 1.60]	0.59
	Headache days				2 months after randomization	2 (240)	43%	REM	MD	−0.47	[−2.31, 1.36]	0.61
					3-4 months after randomization	2 (238)	21%	REM	MD	−0.12	[−1.41, 1.17]	0.86
					5-6 months after randomization	2 (233)	38%	REM	MD	−0.07	[−1.66, 1.52]	0.93
	Analgesic use				2 months after randomization	5 (368)	0%	REM	SMD	−0.08	[−0.29, 0.14]	0.50
					3-4 months after randomization	7 (455)	74%	REM	SMD	−0.27	[−0.71, 0.16]	0.21
					5-6 months after randomization	6 (410)	94%	REM	SMD	−0.56	[−1.57, 0.46]	0.28
					>6 months after randomization	5 (171)	0%	REM	SMD	0.02	[−0.30, 0.35]	0.89
	Headache frequency	Preventive treatment	Acupuncture	③	2 months after randomization	3 (553)	0%	REM	SMD	−0.24	[−0.40, −0.08]	0.003
					3-4 months after randomization	4 (780)	0%	REM	SMD	−0.26	[−0.41, −0.11]	0.00008
					5-6 months after randomization	3 (714)	0%	REM	SMD	−0.20	[−0.35, −0.05]	0.010
	Migraine attacks				2 months after randomization	2 (241)	0%	REM	MD	−0.49	[−0.91, −0.08]	0.020
					3-4 months after randomization	3 (316)	0%	REM	MD	−0.32	[−0.59, −0.04]	0.025
					5-6 months after randomization	2 (237)	78%	REM	MD	−0.47	[−1.22, 0.28]	0.22
	Migraine days				2 months after randomization	2 (503)	0%	REM	MD	−0.58	[−1.09, −0.07]	0.025
					3-4 months after randomization	2 (553)	0%	REM	MD	−0.70	[−1.23, −0.17]	0.0090
					5-6 months after randomization	2 (564)	0%	REM	MD	−0.66	[−1.18, −0.13]	0.014
	Analgesic use				2 months after randomization	2 (241)	28%	REM	SMD	−0.22	[−0.53, 0.08]	0.15
					3-4 months after randomization	2 (239)	0%	REM	SMD	−0.08	[−0.33, 0.18]	0.56
					5-6 months after randomization	2 (237)	0%	REM	SMD	−0.09	[−0.35, 0.17]	0.49

Linde et al. [24]	Headache frequency			①	Median 3 months	4 (1199)	7%	FEM	SMD	−0.56	[−0.65, −0.48]	<0.00001
				②	Median 3 months	12 (1646)	47%	FEM	SMD	−0.18	[−0.28, −0.08]	0.0004
					Median 12 months	9 (1534)	59%	FEM	SMD	−0.19	[−0.30, −0.09]	0.0003
				③	Median 3 months	3 (739)	0%	FEM	SMD	−0.25	[−0.39, −0.10]	0.001
					Median 12 months	3 (744)	0%	FEM	SMD	−0.13	[−0.28, 0.01]	0.08

Gao et al. [20]	Headache days	NR	Acupuncture	②	After treatment	3 (775)	45%	FEM	MD	0.18	[−0.38, 0.73]	0.54
					After follow-up	3 (839)	59%	FEM	MD	−0.17	[−0.69, 0.35]	0.53

Pu [24]	Migraine days	Preventive treatment	Acupuncture	③	3-4 months after randomization	4 (755)	3%	FEM	SMD	−0.30	[−0.45, −0.16]	<0.0001
					5-6 months after randomization	2 (564)	0%	FEM	SMD	−0.66	[−1.18, −0.13]	0.01
	Migraine frequency				3-4 months after randomization	3 (316)	0%	FEM	MD	−0.32	[−0.59, −0.04]	0.03
					5-6 months after randomization	2 (237)	78%	REM	MD	−0.47	[−1.22, 0.28]	0.22
	Analgesic use				3-4 months after randomization	4 (388)	83%	REM	SMD	−0.34	[−0.85, 0.17]	0.20
					5-6 months after randomization	3 (321)	0%	FEM	SMD	−0.22	[−0.44, 0.00]	0.06

Song et al. [26]	Headache times	Preventive treatment	Acupuncture with or without placebo	③ with or without placebo	Long-term (ND)	2 (164)	42%	FEM	MD	−0.79	[−1.39, −0.20]	0.009

^*∗*^NR: not reported; ND: not defined; ①: no acupuncture; ②: sham acupuncture; ③: drug treatment; FEM: fixed effects model; REM: random effects model.

**Table 6:** MAs outcomes about efficacy.

Study ID	Outcome	Acute or preventive treatment	Intervention	Control	Follow-up time	RCTs (*n*)	I2	Model	Scale	Effect	95% CI	*P* value
Linde et al. [14]	Response (attack frequency reduction of at least 50%)	ND	Acupuncture	①	3-4 months after randomization	4 (2376)	0%	REM	RR	2.33	[2.02, 2.69]	<0.00001
				②	2 months after randomization	7 (1091)	59%	REM	RR	1.38	[0.96, 1.97]	0.08
					3-4 months after randomization	11 (1225)	14%	REM	RR	1.13	[0.95, 1.35]	0.16
					5-6 months after randomization	6 (1054)	64%	REM	RR	1.19	[0.86, 1.63]	0.29
					>6 months after randomization	3 (132)	10%	REM	RR	1.01	[0.51, 1.99]	0.98
				③	2 months after randomization	2 (566)	0%	REM	RR	1.35	[1.09, 1.67]	0.006
					3-4 months after randomization	2 (566)	0%	REM	RR	1.20	[0.98, 1.46]	0.08
					5-6 months after randomization	2 (564)	62%	REM	RR	1.35	[0.89, 2.05]	0.16

Linde et al. [7]	Response (at least 50% frequency reduction)	Preventive treatment	Acupuncture	①	Median 3 months	4 (2519)	7%	FEM	RR	2.40	[2.08, 2.76]	<0.00001
				②	Median 3 months	14 (1825)	48%	FEM	RR	1.23	[1.11, 1.36]	<0.00001
					Median 12 months	11 (1683)	61%	FEM	RR	1.25	[1.13, 1.39]	<0.00001
				③	Median 3 months	3 (743)	0%	FEM	RR	1.24	[1.08, 1.44]	0.003
					Median 12 months	3 (744)	0%	FEM	RR	1.11	[0.97, 1.26]	0.12

Chen et al. [15]	Effective rate (headache relieved or disappeared)	NR	Acupuncture	③	ND	18 (1730)	0%	FEM	OR	5.11	[3.81, 6.85]	<0.00001

Chen [16]	Effective rate (headache controlled or disappeared)	NR	Acupuncture	③	ND	18 (1606)	42%	FEM	RR	1.21	[1.16, 1.27]	<0.00001

Chu et al. [17]	Effective rate (ND)	NR	Acupuncture	②	ND	2 (139)	0%	FEM	OR	5.70	[1.88, 17.27]	0.002
				③	ND	20 (2096)	13%	FEM	OR	3.96	[3.02, 5.19]	<0.00001
				TCM drug treatment	ND	2 (162)	0%	FEM	OR	7.58	[2.30, 24.92]	0.0009

Dai and Lin [19]	Cure rate (ND)	NR	Acupuncture	③	ND	2 (140)	0%	FEM	OR	3.02	[1.00, 9.07]	0.05
	Response rate (include cure rate)	NR			ND	2 (140)	0%	FEM	OR	5.37	[2.57, 11.21]	<0.00001
	Effective rate (cure and response rates)	NR			ND	2 (140)	0%	FEM	OR	4.85	[1.69, 13.94]	0.003
Gao et al. [20]	Effective rate (at least 50% frequency reduction)	NR	Acupuncture	②	ND	8 (1253)	0%	FEM	OR	1.28	[1.02, 1.61]	0.03

Zheng and Hai [22]	Effective rate (ND)	NR	Acupuncture	②	ND	3 (217)	69%	REM	RR	1.87	[1.17, 2.98]	0.009
				③	ND	28 (3059)	81%	REM	RR	1.24	[1.16, 1.33]	<0.00001
				TCM drug treatment	ND	3 (225)	0%	FEM	RR	1.29	[1.14, 1.45]	<0.00001

Zhao [23]	Effective rate (ND)	NR	Acupuncture	③	ND	8 (686)	50%	REM	RR	1.19	[1.07, 1.32]	0.0010
			Acupuncture with acupuncture on ear	③	3 months	2 (152)	0%	FEM	RR	1.18	[1.05, 1.33]	0.005
			Acupuncture with TCM drug treatment	③	At least 6 months	3 (257)	98%	REM	RR	1.24	[0.61, 2.52]	0.0009

Pu [24]	Effective rate (at least 50% frequency reduction)	Preventive treatment	Acupuncture	③	3-4 months after randomization	4 (772)	57%	REM	RR	1.41	[1.06, 1.88]	0.02
					5-6 months after randomization	2 (564)	0%	FEM	RR	1.18	[0.97, 1.43]	0.11

Song et al. [26]	Effective rate (ND)	Preventive treatment	Acupuncture	③	1-3 months	15 (1218)	35%	FEM	OR	2.76	[2.03, 3.77]	<0.00001
					>3 months	7 (642)	0%	FEM	OR	4.17	[2.80, 6.20]	<0.00001

Yang et al. [27]	Efficacy	NR	Acupuncture	③	Short-term (ND)	9 (821)	69%	REM	RR	1.27	[1.11, 1.45]	0.0004
					Long-term (ND)	4 (232)	87%	REM	RR	1.76	[1.05, 2.94]	0.03

^*∗*^NR: not reported; ND: not defined; ①: no acupuncture; ②: sham acupuncture; ③: drug treatment; FEM: fixed effects model; REM: random effects model.

**Table 7:** MAs outcomes about adverse events.

Study ID	Outcome	Acute or preventive treatment	Intervention	Control	Follow-up time	RCTs (*n*)	I2	Model	Scale	Effect	95% CI	*P* value
Linde et al. [14]	Number of patients reporting adverse effects	ND	Acupuncture	②	NR	4 (838)	73%	FEM	OR	0.47	[0.34, 0.65]	<0.00001
	Number of patients dropping out due to adverse effects				NR	2 (191)	0%	FEM	OR	0.10	[0.01, 0.78]	0.028

Linde et al. [7]	Number of participants dropping out due to adverse effects	Preventive treatment	Acupuncture	①	NR	7 (931)	0%	FEM	OR	2.84	[0.43, 18.71]	0.28
	Number of participants reporting adverse effects					4 (1414)	0%	FEM	OR	1.15	[0.85, 1.56]	0.35
	Number of participants with serious adverse events					6 (1071)	0%	FEM	OR	1.29	[0.43, 3.83]	0.65
	Number of participants dropping out due to adverse effects			②	NR	4 (451)	0%	REM	OR	0.27	[0.08, 0.86]	0.027
	Number of participants reporting adverse effects					5 (931)	78%	REM	OR	0.25	[0.10, 0.62]	0.0027
	Number of participants with serious adverse events					3 (721)	0%	REM	OR	1.33	[0.38, 4.73]	0.66

Chen [16]	Number of adverse effects	ND	Acupuncture	ND	NR	5 (567)	0%	FEM	RR	0.11	[0.03, 0.49]	0.003

Pu [24]	Rate of adverse events	Preventive treatment	Acupuncture	②	NR	7 (1126)	77%	REM	RR	0.33	[0.17, 0.64]	0.001

^*∗*^NR: not reported; ND: not defined; ①: sham acupuncture; ②: drug treatment; FEM: fixed effects model; REM: random effects model.
